# Clinical Applications of Natriuretic Peptides in Assessment of Valvular Heart Disease

**DOI:** 10.1155/2015/807861

**Published:** 2015-07-22

**Authors:** Abhishek Sharma, Vaseem Ahmed, Aakash Garg, Chirag Aggarwal

**Affiliations:** ^1^Division of Cardiovascular Diseases, State University of New York, Downstate Medical Center, Brooklyn, New York, NY 1203, USA; ^2^Department of Medicine, Richmond University Medical Center, Staten Island, New York, NY 10310, USA; ^3^Department of Medicine, Saint Peter's University Hospital, New Brunswick, NJ 08901, USA; ^4^Division of Cardiology, Icahn School of Medicine at Mount Sinai, New York, NY 1030, USA

## Abstract

Biomarkers such as natriuretic peptides (NPs) have evolving clinical utility beyond the scope of heart failure. The role of NPs in the management of valvular heart disease is a growing area of investigation. NPs have much potential in the assessment of asymptomatic patients with hemodynamically significant valvular lesions who have traditionally been excluded from consideration of surgical intervention. NPs also have a role in the risk stratification of these patients as well as in routine surveillance and monitoring. Together with echocardiographic data and functional status, NPs are being incorporated into the management of valvular heart disease. In this review we examine the evidence for the role of natriuretic peptides in assessment of VHD.

## 1. Introduction

Valvular heart disease (VHD) is comprised primarily of stenotic and regurgitant lesions of heart valves. VHD is among the most common causes of heart failure and sudden cardiac death [1, 2]. It is known to be a progressive disease and a high proportion of asymptomatic patients have hemodynamically apparent VHD on cardiovascular imaging [2]. Age related detrimental changes in valve structure and physiology are major determinants in the development of valvular heart disease and many patients belong to the elderly population. The burden of VHD is expected to increase in the future primarily due to the aging population and increasing life expectancy. As such, there is significant economic and public health interest in the management and potential for early identification of VHD.

Echocardiography is the most commonly used imaging modality to assess the severity of VHD and left ventricular (LV) function; however it is operator dependent and has several limitations. For this reason, there is a growing need for other objective measures to help identify patients with VHD. Various biomarkers have been examined for diagnosis and assessment of VHD severity and preoperative risk stratification. The list of such biomarkers is long and includes but is not limited to brain natriuretic peptide (BNP), NT-BNP (N-terminal part of BNP), N-terminal-pro-BNP (NT-pro-BNP), cardiotrophin-1, and tumor necrosis factor receptors 1 and 2. For a biomarker to be used in assessment and management of VHDs, it must reflect LV wall stress and predict echocardiographic and clinical progression of the disease. Among the various biomarkers, natriuretic peptides have shown promising results in several studies, and BNP in particular has emerged as an important potential biomarker for the diagnosis, management, and assessment of valvular heart diseases. In this review we examine the evidence for the role of natriuretic peptides in assessment of VHD.

## 2. Physiology of BNP in Heart Failure

Atrial natriuretic peptide (ANP) and B-type natriuretic peptide are the two major hormones which are produced by cardiac myocytes in response to increased stress in the form of either pressure or volume overload. ANP is generally stored in the atrial muscles and released in response to stretch, whereas BNP is released by muscle tissue of the ventricles. BNP is a prohormone which is cleaved to produce its inactive form known as NT-pro-BNP [3, 4]. They both exert diuretic, natriuretic, and hypotensive effects by inhibition of renin-angiotensin system and sympathetic activity [5]. Assays are readily available in most hospital laboratories and measure both BNP and NT-pro-BNP. The normal plasma concentration of NT-pro-BNP tends to be higher than BNP by as much as 10-fold [6] given the former has a longer half-life [7] of 1-2 hours as compared to the half-life of BNP being approximately 22 minutes [3]. A normal level of natriuretic peptides can be difficult to define as there are many variables affecting each biomarker in different individuals. The two increase with age, in female gender [8], and with worsening renal function, particularly NT-pro-BNP [9]. BNP is a quick diagnostic test that is not used alone but can support a clinician's diagnosis of decompensated heart failure and help identify the etiology of a patient's dyspnea [10].

## 3. Role of Natriuretic Peptides in Assessment of Patients with Various VHDs

### 3.1. Aortic Stenosis

NPs are shown to be correlated with the severity of aortic stenosis (AS) measured either by transvalvular gradient or valvular area [11–17]. Gerber and colleagues evaluated the relationship between natriuretic peptide concentration in patients with isolated aortic stenosis (peak aortic velocity of ≥2.5 m/s) with severity of AS and the presence of symptoms [15]. The result showed a positive correlation between the higher values of BNP, NT-pro-BNP, and ANP with the severity of AS; LV and left atrium size, LV afterload represented by end-systolic wall stress wall stress, and thickness; and right ventricular pressures [15]. The values of NPs were highest especially in cases where ejection fraction was less than 50% and values increased steadily with decreased function of the left ventricle [15]. Out of all the NPs examined, NT-pro-BNP correlated most extensively with the deterioration of LV function with a cut-off value of 60 pmol/litre [15]. The levels of NP showed positive correlation with the degree LV hypertrophy and a negative correlation with LV ejection fraction [11, 15, 18].

Lim et al. evaluated prognostic value of serum BNP concentration in patients with isolated severe AS (aortic valve area < 1 cm^2^) and preserved LV function [19]. Serum BNP level > 66 pg/mL has been shown to be a useful screening tool to differentiate symptomatic and asymptomatic patients [19]. They proposed use of serum BNP levels as an alternative/complementary to exercise testing in patients with severe AS who have equivocal symptoms [19]. Further, serum BNP levels have also been shown to stratify AS patients for future adverse cardiovascular outcomes [19]. Similarly, in a prospective study of 130 patients with severe AS (peak velocity > 4 m/s and/or aortic valve area < 1.0 cm^2^), NT-BNP has been reported to provide valuable prognostic information and predict postoperative outcomes beyond clinical and echocardiographic evaluation [20].

Of particular interest is the expanding evidence for prognostic role of NPs after transcatheter aortic valve replacement (TAVR) for AS [21, 22]. As the wall stress would be expected to reduce after successful TAVR, postprocedure AR can contribute to continued LV strain and release of BNP and NT-pro-BNP. In this context, serial measurements of NPs can provide substantial insight into postprocedure outcomes [21]. In a subanalysis from PARTNER trial, increase in 30-day post-TAVR BNP levels compared with baseline was significantly associated with higher one-year mortality rates [23].

### 3.2. Aortic Regurgitation

Aortic regurgitation (AR) is a valvulopathy associated with considerable controversy in regard to the timing of intervention. As per current guidelines of the American College of Cardiology/American Heart Association (ACC/AHA), the presence of symptoms is a class I indication for surgical intervention. Asymptomatic patients with an EF of less than 50% or those who are requiring other cardiac surgeries also meet this class I indication for surgical replacement [24]. However, a subset of patients with asymptomatic but hemodynamically severe AR and preserved ejection fraction (>55%) has no clear recommendations for intervention. Prior studies have shown that effective regurgitant orifice area (EROA), an echocardiographic parameter, is associated with poorer outcomes [25]. Additionally, BNP is a relatively newer entity being investigated in asymptomatic patients with AR [26]. Previously, it was shown that asymptomatic patients with AR and preserved ejection fraction have elevated serum BNP levels [27].

Pizarro et al. prospectively investigated 294 patients with asymptomatic severe AR and preserved EF to determine an additive prognostic role of BNP levels in risk stratifying of patients into a high risk category [28]. Their results suggested an incremental number of adverse outcomes such as decompensated heart failure, LV dysfunction, and death, with the cut-off value of BNP greater than 130 pg/mL. A plausible explanation for these results could be the direct correlation between BNP levels and severity of aortic regurgitation [26]. Furthermore, the role of NT-pro-BNP as a marker of severity and surveillance tool for follow-up has been validated in a prospective study [29]. Weber and colleagues have demonstrated a decrease in levels of BNP and NT-pro-BNP after surgical valve replacement for AR [29]. On the contrary, levels increased in patients managed conservatively, supporting the link between disease progression and worsened LV wall stress.

These findings therefore point to an independent prognostic value of BNP which could complement other echocardiographic parameters, in this subset of patients.

### 3.3. Mitral Regurgitation

Mitral regurgitation (MR) is a common valvular disease in the western population. At present, the guideline-recommended indications for surgery are (a) severe symptomatic MR or (b) asymptomatic chronic severe MR and mild to moderate LV dysfunction, ejection fraction 0.30 to 0.60, and/or end-systolic dimension greater than or equal to 40 mm [24]. As is the case with aortic valve disease, the timing of surgery in mitral regurgitation is a very crucial and challenging decision to make. The common opinion is to consider surgery before appearance of symptomatology [30].

Mitral regurgitation (MR) has been further classified as chronic primary (degenerative) MR and chronic secondary (functional) MR [30]. Symptomatology is an ACC/AHA class I indication for intervention in these patients; however the challenge arises in assessing the presence of symptoms due to the subjective nature of this identification. In addition to determining optimal time of surgical intervention, biomarkers such as NT-pro-BNP are important in risk stratification of patients [31]. Traditionally, transthoracic echocardiography at both rest and stress has been used in risk stratification of patients prior to intervention; however integration of biomarkers with imaging data continues to be investigated [32].

Pizarro et al. studied patients with isolated severe asymptomatic organic MR with normal LV function defined as ejection fraction of greater than 60% [33]. Inclusion criterion was preserved exercise tolerance as determined by Bruce-protocoled exercise EKG testing with functional capacity of more than 7 metabolic equivalents of task (METs) and without any symptoms or significant electrocardiographic aberrations. The results concluded that patients with a baseline BNP of greater than 105 pg/mL had worse outcomes at one year, conferring an independent prognostic utility of BNP in patients with asymptomatic severe MR. Further, BNP levels were even stronger predictors than various echocardiographic parameters such as effective orifice regurgitant area (EROA) and left ventricular end-systolic diameter [32]. Henceforth, it was suggested that BNP could be used in conjunction with traditional imaging modalities to risk-stratify asymptomatic patients who may benefit from earlier intervention.

While the above study examined BNP levels at rest, Magne et al. sought to evaluate the prognostic value of BNP levels during exercise. Similarly, in asymptomatic subjects with MR, they found elevated exercise BNP to be independently associated with a higher risk of adverse cardiac events at follow-up [34]. The study proposed an additional value of exercise BNP in prognosis and risk stratification beyond the resting BNP for the evaluation of early mitral valve intervention.

In a population based survey of elderly patients, significant increases in levels of N-terminal ANP were found with mitral regurgitation [35]. Kerr et al. demonstrated increment in levels of BNP in asymptomatic and mildly symptomatic patients with moderate to severe MR [32]. The study however was unable to predict severity of MR on the basis of BNP values. Similarly, Detaint et al. demonstrated that, in patients with chronic organic MR, high BNP levels were independently associated with worse CV outcomes and predicted atrial and ventricular remodelling but did not correlate with degree of MR [36, 37], whereas Sutton et al. showed a positive correlation of BNP, NT-pro-BNP, and ANP with severity of mitral regurgitation and left atrial dimensions [38]. Among the aforementioned NPs, NT-pro-BNP showed strongest correlation. Thus, while studies support the prognostic role of NPs in assessment and risk stratification of patients with organic MR, the correlation between MR severity and BNP levels still remains to be established.

Moreover, there is paucity of data evaluating role of BNP or NT-pro-BNP in patients undergoing transcatheter mitral valve repair or replacement (TMVR). In subanalysis of MitraSWISS registry, Toggweiler and colleagues have reported significantly lower survival rates among patients who have NT-pro-BNP ≥ 5000 ng/L at baseline [HR 6.18 (95% CI 1.84 to 20.79, *p* < 0.01)] on univariate analysis after 2 years of MitraClip therapy [39]. Although significant, results of this study should be interpreted with caution, due to small sample size and lack of multivariate analysis [39]. Thus, though NPs have potential to be a promising tool in management of MR, their role still needs to be evaluated in larger studies before supporting their routine use in patients with MR especially after TMVR.

### 3.4. Mitral Stenosis

Mitral stenosis (MS) is most commonly caused by rheumatic fever; however, its incidence has declined considerably over the past several decades [40]. The cardiovascular implications are significant as patients become symptomatic later in adulthood. The role of biomarkers such as NPs in MS has been examined in several studies, suggesting that plasma BNP levels correlate directly with mitral valve disease severity [41, 42]. Current ACC/AHA guidelines define “severe” MS as mitral valve area ≤ 1.5 cm^2^ or the time at which a patient becomes symptomatic. These criteria are also the class I indications for intervention, more specifically percutaneous mitral balloon commissurotomy [24]. Surgical repair or replacement is reserved for those patients who have failed or were not candidates for percutaneous valvotomy.

Sharma et al. in 2011 performed a study to compare atrial (ANP) and brain natriuretic peptides in the assessment of mitral stenosis and its severity [42]. They discovered that ANP and BNP levels were closely correlated and that BNP was a stronger predictor for reduced exercise capacity as assessed by exercise treadmill testing. The investigators asserted that New York Heart Association functional classification status was insufficient in predicting exercise capacity and that BNP could provide more valuable information. The study also showed that patients with BNP of greater than 84 pg/mL were more likely to have decreased exercise tolerance and a higher rate of adverse outcomes during the follow-up period, leading to a higher rate of intervention [42]. This finding has important clinical utility as it may help clinicians identify high risk patients earlier on. By measuring BNP levels in asymptomatic patients with MS, hemodynamically significant lesions may be identified earlier on and potentially reduce the occurrence of adverse events such as heart failure and number or frequency of hospitalizations.

A Turkish study examined NT-pro-BNP to determine if and how closely the biomarker correlated with clinical symptoms and echocardiographic evidence of MS. They found that significantly higher levels of NT-pro-BNP had a close correlation with echocardiographic data and functional capacity of patients [43]. The findings have important clinical utility as they suggest that the noninvasive and more economical diagnostic testing of NT-pro-BNP may allow for easier and less labor intensive surveillance of MS. Further, biomarker testing could be of particular benefit in patients whose body habitus does not allow for favorable echocardiographic images.

As mentioned previously, percutaneous balloon valvuloplasty is the surgical intervention that is typically used to manage patients with MS and meet the indication for intervention. BNP and NT-pro-BNP levels have been repeatedly shown to decline after successful intervention [44–47]. This has the potential for clinicians to serially monitor NP levels following surgery to assess for success of the procedure improvement in the stenotic lesion. Interestingly, Razzolini et al. examined NPs before and after percutaneous intervention and found that NP levels were not significantly impacted in patients with atrial fibrillation [48]. They found that NPs declined after surgery only in patients in sinus rhythm at the time of the procedure, suggesting that the ideal time to operate is while patients are in sinus rhythm.

### 3.5. Limitations of Natriuretic Peptides

NPs are a helpful tool to aid in the evaluation of asymptomatic patients with hemodynamically significant valvulopathy as well as in the surveillance of known valvular disease to monitor disease progression. There are however limitations that should be noted when using BNP and NT-pro-BNP clinically. NPs have been traditionally used in the inpatient setting to support the clinical diagnosis of decompensated heart failure [49]. The mechanism for this is related to the ventricular wall stress caused by volume and pressure overload leading to BNP release by cardiac myocytes in response to stretch. However there are clinical scenarios other than heart failure that can lead to an increase in BNP. Some of these include renal dysfunction, acute coronary syndromes, advanced age, acute illness, prior lung disease, acute pulmonary embolism, and atrial fibrillation [3, 4]. Similarly, multiple coexisting valvular lesions are not uncommon and can also cause further elevation of NPs and contribute to confounding factors in their interpretation. Further, the coefficient of variation (CV) in intra-assay precision for commonly used BNP assay has been reported to vary from 5 to 15% [50–52]. Therefore when examining a patient's BNP or NT-pro-BNP level it is important to consider all potential causes for their elevation and assess the patient carefully.

Just as there are clinical conditions which drive BNP levels up further, there are also conditions which cause BNP to be lower than expected. One commonly known reason for this is obesity and while the exact mechanism is unclear, there is some speculation as to why. One explanation suggests that adipocytes express higher levels of clearance receptors leading to lower plasma concentrations [40]. Other conditions demonstrating lower than expected BNP levels include flash pulmonary edema, tamponade, and constrictive pericarditis [3].

Another potential limitation to the clinical application and value of BNP is its variation between individuals. Certainly the aforementioned conditions causing fluctuations in BNP can account for some variation between patients; however it is important to determine a patient's own baseline BNP or NT-pro-BNP level to more accurately identify significant changes [3]. These biomarkers can be measured in both the inpatient and outpatient setting and can aid in the determination of a patient's volume status. By being able to identify a patient's baseline natriuretic peptide level when they are euvolumic, clinicians can use these biomarkers in other clinical applications such as in the assessment of valvular heart disease without the confounding aspect of the biomarkers. The patient's baseline biomarker also allows more individualized management for patients.

In addition to variation between individuals there can be considerable variation within an individual, particularly in a patient with chronic heart failure. Although there is conflicting data, patients generally require a change in NT-pro-BNP and BNP of 25% and 40%, respectively, to represent a clinically significant change in volume status [53, 54]. This must also be taken into consideration when interpreting natriuretic peptide levels for purposes other than assessment of heart failure.

## 4. Conclusion

The measurement of biomarkers such as BNP and NT-pro-BNP is readily accessible diagnostic aids and provide valuable information in risk stratification and postprocedure outcomes in patients with VHD. These biomarkers are particularly helpful in the early identification of patients with hemodynamically significant valvulopathy in the absence of clinical symptoms. Further, the emergence of BNP as an independent prognostic biomarker for the evaluation of valvular heart disease highlights the need for further research regarding early surgical intervention in specific patient populations, particular those with asymptomatic but severe lesions.
